# In Vivo Availability of Cannabinoid 1 Receptor Levels in Patients With First-Episode Psychosis

**DOI:** 10.1001/jamapsychiatry.2019.1427

**Published:** 2019-07-03

**Authors:** Faith Borgan, Heikki Laurikainen, Mattia Veronese, Tiago Reis Marques, Merja Haaparanta-Solin, Olof Solin, Tarik Dahoun, Maria Rogdaki, Raimo KR Salokangas, Max Karukivi, Marta Di Forti, Federico Turkheimer, Jarmo Hietala, Oliver Howes

**Affiliations:** 1 Psychosis Studies Department, Institute of Psychiatry, Psychology and Neuroscience, King’s College London, London, United Kingdom; 2 MRC London Institute of Medical Sciences, Faculty of Medicine, Imperial College London, London, United Kingdom; 3 Turku PET (Positron Emission Tomography) Centre, University of Turku and Turku University Hospital, Turku, Finland.; 4 Department of Psychiatry, University of Turku and Turku University Hospital, Turku, Finland.; 5 Centre for Neuroimaging Sciences, Institute of Psychiatry, Psychology and Neuroscience, King’s College London, London, United Kingdom; 6 Institute of Clinical Sciences, Faculty of Medicine, Imperial College London, Hammersmith Hospital, London, United Kingdom; 7 Department of Psychiatry, University of Oxford, Warneford Hospital, Oxford, United Kingdom; 8 Department of Psychiatry, Turku University, Satakunta Hospital District, Turku, Finland

## Abstract

**Importance:**

Experimental and epidemiological studies implicate the cannabinoid 1 receptor (CB1R) in the pathophysiology of psychosis. However, whether CB1R levels are altered in the early stages of psychosis and whether they are linked to cognitive function or symptom severity remain unknown.

**Objective:**

To investigate CB1R availability in first-episode psychosis (FEP) without the confounds of illness chronicity or the use of illicit substances or antipsychotics.

**Design, Setting, and Participants:**

This cross-sectional, case-control study of 2 independent samples included participants receiving psychiatric early intervention services at 2 independent centers in Turku, Finland (study 1) and London, United Kingdom (study 2). Study 1 consisted of 18 volunteers, including 7 patients with affective or nonaffective psychoses taking antipsychotic medication and 11 matched controls; study 2, 40 volunteers, including 20 antipsychotic-naive or antipsychotic-free patients with schizophrenia or schizoaffective disorder and 20 matched controls. Data were collected from January 5, 2015, through September 26, 2018, and analyzed from June 20, 2016, through February 12, 2019.

**Main Outcomes and Measures:**

The availability of CB1R was indexed using the distribution volume (V_T_, in milliliters per cubic centimeter) of 2 CB1R-selective positron emission tomography radiotracers: fluoride 18–labeled FMPEP-d_2_ (study 1) and carbon 11–labeled MePPEP (study 2). Cognitive function was measured using the Wechsler Digit Symbol Coding Test. Symptom severity was measured using the Brief Psychiatric Rating Scale for study 1 and the Positive and Negative Syndrome Scale for study 2.

**Results:**

A total of 58 male individuals were included in the analyses (mean [SD] age of controls, 27.16 [5.93] years; mean [SD] age of patients, 26.96 [4.55] years). In study 1, 7 male patients with FEP (mean [SD] age, 26.80 [5.40] years) were compared with 11 matched controls (mean [SD] age, 27.18 [5.86] years); in study 2, 20 male patients with FEP (mean [SD] age, 27.00 [5.06] years) were compared with 20 matched controls (mean [SD] age, 27.15 [6.12] years). In study 1, a significant main effect of group on [^18^F]FMPEP-d_2_ V_T_ was found in the anterior cingulate cortex (ACC) (*t*_16_ = −4.48; *P* < .001; Hedges *g* = 1.2), hippocampus (*t*_16_ = −2.98; *P* = .006; Hedges *g* = 1.4), striatum (*t*_16_ = −4.08; *P* = .001; Hedges *g* = 1.9), and thalamus (*t*_16_ = −4.67; *P* < .001; Hedges *g* = 1.4). In study 2, a significant main effect of group on [^11^C]MePPEP V_T_ was found in the ACC (Hedges *g* = 0.8), hippocampus (Hedges *g* = 0.5), striatum (Hedges *g* = 0.4), and thalamus (Hedges *g* = 0.7). In patients, [^11^C]MePPEP V_T_ in the ACC was positively associated with cognitive functioning (*R* = 0.60; *P* = .01), and [^11^C]MePPEP V_T_ in the hippocampus was inversely associated with Positive and Negative Syndrome Scale total symptom severity (*R* = −0.50; *P* = .02).

**Conclusions and Relevance:**

The availability of CB1R was lower in antipsychotic-treated and untreated cohorts relative to matched controls. Exploratory analyses indicated that greater reductions in CB1R levels were associated with greater symptom severity and poorer cognitive functioning in male patients. These findings suggest that CB1R may be a potential target for the treatment of psychotic disorders.

## Key Points

**Question:**

Is the cannabinoid 1 receptor altered in first-episode psychosis without the confounds of cannabis use and illness chronicity?

**Findings:**

In this cross-sectional case-control study of 58 male individuals, cannabinoid 1 receptor availability was lower in male patients with first-episode psychosis who did not use cannabis, including in patients who were antipsychotic naive or antipsychotic free, and exploratory analyses indicated that greater reductions are associated with poor cognitive functioning and greater symptom severity.

**Meaning:**

Cannabinoid 1 receptor alterations are evident early in psychosis and may be linked to symptom severity and cognition, which suggests that cannabinoid 1 receptor modulation is a potential target for the treatment of psychotic disorders.

## Teaser

This cross-sectional case-control cohort study investigates availability of cannabinoid 1 receptor in patients with first-episode psychosis without the confounds of substance use, antipsychotic medication use, or illness chronicity.

## Introduction

Schizophrenia and other psychotic disorders affect approximately 1% of the population^1^ and are ranked within the top 10 most disabling health conditions worldwide.^2^ Meta-analytic findings indicate that cannabis use increases the relative risk of psychosis.^3^ The main psychoactive chemical in cannabis, delta-9-tetrahydrocannabinol (THC), acts as a partial cannabinoid 1 receptor (CB1R) agonist.^4^ Short-term use of THC induces psychotic symptoms and cognitive deficits in controls^5,6,7^ and exacerbates these symptoms in patients with schizophrenia.^8^ The most widely studied endogenous CB1R agonist, anandamide (AEA), is also elevated in vivo in cerebrospinal fluid in individuals at risk for psychosis^9^ and in patients with first-episode psychosis (FEP) who have not received medication and who do not use cannabis.^10,11^

Cannabinoid 1 receptors are G-protein–coupled receptors expressed on presynaptic nerve terminals of excitatory and inhibitory neurons throughout the cortex, thalamus, hippocampus, and striatum.^12^ Ex vivo studies in schizophrenia have reported lower CB1R messenger RNA and protein levels^13,14,15^ but higher CB1R density.^16,17,18,19^ In vivo studies in schizophrenia have also reported higher^20,21^ and lower^22^ CB1R availability. Although higher levels were reported in vivo when no arterial blood sampling was used,^20^ arterial blood sampling is needed to estimate the proportion of the radiotracer that is available to enter the brain.^23^ Although higher levels were also reported in the pons (N = 9) when using arterial blood sampling,^21^ a larger study using the same radiotracer with arterial blood sampling reported lower CB1R availability (N = 25).^22^ However, both studies^21,22^ included patients with chronic schizophrenia who were receiving antipsychotics, and in some cases the exclusion of cannabis use or dependence was unclear.^21^

We therefore investigated in vivo CB1R availability in 2 independent cohorts of patients with FEP. Given the findings from the largest in vivo study,^22^ we hypothesized that patients would show lower CB1R availability relative to matched controls. Because previous work has shown that CB1R agonists induce cognitive impairments,^5,6,8^ exploratory analyses investigated the association between CB1R availability and cognition.

## Methods

### Ethics Statement

Ethical approvals were obtained from the study sites in Turku, Finland, and London, United Kingdom. Volunteers demonstrated capacity and provided informed written consent. We followed the Strengthening the Reporting of Observational Studies in Epidemiology (STROBE) reporting guideline for case-control studies.

For study 1, 14 patients were screened for eligibility, 13 were deemed eligible, and 7 were included in the study; and 25 healthy volunteers were screened for eligibility, 13 were deemed eligible, and 11 were included in the study. The overall number of patients and controls that were considered for inclusion was not recorded for study 1. For study 2, clinical teams indicated that approximately 400 patients (3% of 125 000 patients seen during a 4-year period by clinical teams) were potentially eligible, 115 patients were examined for eligibility, and 106 patients were deemed eligible and included in the study. However, 66 of 106 patients (62.3%) were later withdrawn from the study owing to a loss of capacity to consent, hospital admission, or the commencement of antipsychotic treatment. A total of 40 patients were included in the study. We identified 300 potentially eligible healthy volunteers, of whom 40 were deemed eligible and were included in the study.

### Design

Cannabinoid 1 receptor availability was investigated at 2 positron emission tomography (PET) centers using independent samples. Availability of CB1R was indexed using the distribution volume (V_T_) of fluoride 18–labeled FMPEP-d_2_ ([3R,5R]-5-[3-methoxy-phenyl]-3-[{R}-1-phenyl-ethylamino]-1-[4-trifluoro-methyl-phenyl]-pyrrolidin-2-one) (study 1) and carbon 11–labeled MePPEP ([3R,5R]-5-[{3-[^18^F]fluoromethoxy-d2}phenyl]-3-[{R}-1-phenyl-ethylamino]-1-[4-trifluoromethyl-phenyl]-pyrrolidin-2-one) (study 2). Given sex differences in CB1R availability^24^ and previous discrepant findings, we only investigated men to remove sex as a source of variability, with the view of investigating women in a subsequent study.^25^

### Participants

Data were collected from January 5, 2015, through September 26, 2018. Patients with FEP met the following inclusion criteria: (1) *DSM-IV* diagnosis of a psychotic disorder, determined by the *Structured Clinical Interview of *DSM-IV-TR* Axis I Disorders–Patient Edition*^26^; (2) illness duration of less than 3 years; and (3) male sex. In study 1, volunteers were taking antipsychotics and had diagnoses of affective or nonaffective psychosis (Table 1).^27^ In study 2, volunteers were medication naive or free of all pharmacological treatments for at least 6 months and had diagnoses of schizophrenia or schizoaffective disorder (Table 2). Healthy volunteers had no current or lifetime history of an Axis I disorder as determined by the *Structured Clinical Interview of *DSM-IV-TR* Axis I Disorders–Patient Edition*^26^ and were matched by age (±3 years) and sex (male). Exclusion criteria for all volunteers were (1) current or lifetime history of substance abuse or dependence; (2) substance use within the last month; and (3) positive results for cannabis and other substances on screening toxicology tests (see eMethods 1 in the Supplement for the full exclusion criteria).

**Table 1.** Demographics for Study 1^a^

Characteristic	Healthy Volunteers (n = 11)	Patients With FEP (n = 7)^b^	Statistical Test Result	*df*	*P* Value
Age, y, mean (SD)	27.18 (5.86)	26.8 (5.40)	Independent-samples *t* = −0.13	16	.90
Sex, No. male/female	11/0	7/0	NA	NA	NA
Race/ethnicity, No. white/other	11/0	7/0	NA	NA	NA
Employment, No. full-time/part-time/unemployed/ student/missing	9/0/0/4/0	2/1/0/2/2	χ^2^ = 2.55	2	.28
Educational attainment, No. completed high school/did not complete high school/completed university/missing	1/8/2/0	1/4/0/2	χ^2^ = 1.28	2	.53
Educational attainment after compulsory, mean (SD), y	15.73 (3.17)	13.43 (−1.81)	Independent-samples *t* = −1.81	16	.09
Socioeconomic status, No. high/medium/low/student^c^	3/0/4/4	0/0/5/2	χ^2^ = 1.88	3	.60
Current cannabis use, No. yes/no	0/11	0/7	NA	NA	NA
Current alcohol use, No. yes/no/missing	11/0	6/1	χ^2^ = 1.66	1	.20
Frequency of alcohol use, No. none/<1 per mo/2-4 per mo/2-3 per wk/≥4 per wk	0/3/5/2/1	1/4/1/1/0	χ^2^ = 4.48	4	.35
Quantity of alcohol use, No. consuming 1-2/3-4/5-6/7-9/≥10 drinks containing alcohol per session	4/0/1/4/2	2/2/1/0/2	χ^2^ = 6.10	4	.19
Current tobacco use, No. yes/no	10/1	4/3	χ^2^ = 2.82	1	.09
Cigarettes smoked per day, No. smoking 0/1-2/3-5/6-10/11-15/16-19/ 20-25/26-39/≥40	10/0/0/0/1/0/ 0/0/0	4/0/0/1/0/1/ 1/0/0	χ^2^ = 3.87	3	.28
Prior lifetime cannabis use, No. yes/no	3/8	4/3	χ^2^ = 1.60	1	.21
Lifetime cannabis use, No. using 0/1-5/6-10/11-50/>50 joints	3/5/2/1/0	4/1/0/1/1	χ^2^ = 5.18	4	.27
Diagnosis, No. with schizophrenia/schizophreniform disorder/psychotic mood disorder/delusional disorder	NA	2/2/2/1	NA	NA	NA
Illness duration, mean (SD), mo	NA	5.26 (7.32)	NA	NA	NA
Duration of treatment, mean (SD), mo	NA	1.78 (1.06)	NA	NA	NA
Duration of untreated illness, mean (SD), mo	NA	3.47 (7.54)	NA	NA	NA
Current use of antipsychotics, No. yes/no	NA	7/0	NA	NA	NA
Chlorpromazine equivalent dose, mean (SD), mg/d	NA	82.78 (162.55)	NA	NA	NA
Prior use of antidepressant, No. yes/no/missing	NA	3/3/1	NA	NA	NA
BPRS positive score, mean (SD)^d^	NA	21.00 (7.32)	NA	NA	NA
BPRS negative score, mean (SD)^d^	NA	20.71 (7.30)	NA	NA	NA
BPRS general score, mean (SD)^d^	NA	68.29 (16.57)	NA	NA	NA
BPRS total score, mean (SD)^d^	NA	119.39 (28.32)	NA	NA	NA
PANSS total score, mean (SD)^e^	NA	119.39 (28.32)	NA	NA	NA

Abbreviations: BPRS, Brief Psychiatric Rating Scale; FEP, first-episode psychosis; NA, not applicable; PANSS, Positive and Negative Syndrome Scale.

^a^ Used fluoride 18–labeled FMPEP-d_2_ radiotracer.

^b^ Eight patients were recruited for the study. However, because 1 participant was later excluded because of substance dependence, only 7 were included in the analyses.

^c^ High indicates high-, intermediate-, and lower-grade professionals; medium, small employer, self-employed, and lower technical occupations; and low, sales, routine occupations, or unemployed.

^d^ Scores range from 42 to 92, where higher scores indicated greater symptom severity.

^e^ Scores range from 28 to 91, where higher scores indicated greater symptom severity. Total derived from BPRS scores using the equipercentile method.^27^

**Table 2.** Demographics for Study 2^a^

Characteristic	Healthy Volunteers (n = 20)	Patients With FEP (n = 20)	Statistical Test Result	*df*	*P* Value
Age, mean (SD), y	27.15 (6.12)	27.00 (5.06)	Independent-samples *t* = −0.33	38	.74
Sex, No. male/female	20/0	20/0	NA	NA	NA
Race/ethnicity, No. white/black African or Caribbean/Asian/mixed	7/2/9/2	10/4/5/1	NA	NA	NA
Employment, No. full-time/part-time/unemployed/student/missing	9/1/2/8/0	10/4/5/1/0	χ^2^ = 15.39	11	.17
Educational attainment, No. completed high school/did not complete high school/completed university/missing	2/8/10/0	3/8/7/2	χ^2^ = 3.64	2	.16
Educational attainment after compulsory, mean (SD), y	3.84 (2.09)	2.56 (2.20)	Independent-samples *t* = −1.82	38	.08
Socioeconomic status, No. high/medium/low/student/missing^b^	4/6/6/7/0	3/7/3/4/3	χ^2^ = 7.51	9	.58
Current cannabis use, No. yes/no	0/20	0/20	NA	NA	NA
Current alcohol use, No. yes/no/missing	12/8/0	12/8/2	χ^2^ = 1.66	1	.20
Current alcohol use, mean (SD), U/d^c^	0.88 (0.63)	0.63 (1.46)	χ^2^ = 0.53	36	.60
Current tobacco use, No. yes/no/missing	6/14/0	8/10/2	χ^2^ = 0.85	1	.36
Current tobacco use, mean (SD), No. of cigarettes per day	0.50 (1.32)	2.05 (3.32)	χ^2^ = −1.94	37	.07
Diagnosis, No. schizophrenia/schizoaffective disorder	NA	18/2	NA	NA	NA
Illness duration, mean (SD), mo	NA	22.66 (11.64)	NA	NA	NA
Duration of prior treatment, mean (SD), mo	NA	4.21 (5.44)	NA	NA	NA
Current use of antipsychotics, No. yes/no	NA	0/20	NA	NA	NA
Prior use of antipsychotics, No. yes/no	NA	16/4	NA	NA	NA
Current use of antidepressant, No. yes/no	NA	0/20	NA	NA	NA
Prior use of antidepressant, No. yes/no	NA	5/15	NA	NA	NA
Current use of benzodiazepines, No. yes/no	NA	0/20	NA	NA	NA
Prior use of benzodiazepines, No. yes/no	NA	0/20	NA	NA	NA
Digit Symbol Coding Test score, mean (SD)^d^	80.00 (16.47)	68.11 (19.97)	*t* = 1.94	34	.06
PANSS positive score, mean (SD)^e^	NA	26.95 (17.75)	NA	NA	NA
PANSS negative score, mean (SD)^e^	NA	22.79 (6.96)	NA	NA	NA
PANSS general score, mean (SD)^e^	NA	39.74 (10.77)	NA	NA	NA
PANSS total score, mean (SD)^e^	NA	84.21 (22.10)	NA	NA	NA

Abbreviations: FEP, first-episode psychosis; NA, not applicable; PANSS, Positive and Negative Syndrome Scale.

^a^ Used carbon 11–labeled MePPEP radiotracer.

^b^ High indicates high-, intermediate-, and lower-grade professionals; medium, small employer, self-employed, and lower technical occupations; and low, sales, routine occupations, or unemployed.

^c^ 1 unit defined as 10 mL or 8 g of pure alcohol.

^d^ Scores range from 25 to 102, where lower scores indicate greater cognitive impairment.

^e^ Scores range from 28 to 91, where higher scores indicated greater symptom severity.

### Measures

#### Clinical and Demographic Variables

Current age; age at illness onset; illness duration; ethnicity; and use of alcohol, tobacco, and cannabis were recorded (see eMethods 2 in the Supplement for measures). Clinical symptom severity was determined using the Brief Psychiatric Rating Scale^28^ and the Positive and Negative Syndrome Scale^29^ for studies 1 and 2, respectively. Psychotropic medication histories were recorded, and chlorpromazine equivalent doses were calculated using methods described previously.^30^ Cognitive functioning was assessed using the Wechsler Digit Symbol Coding Test^31^ because it is highly correlated with overall cognitive impairments in FEP.^31^

#### Neuroimaging

##### Study 1

Dynamic PET scans were acquired for 0 to 60 and 90 to 120 minutes after a bolus injection of [^18^F]FMPEP-d_2_ (mean [SD], 201 [11.1] MBq) using a high-resolution research tomograph scanner (ECAT; Siemens) in 3-dimensional (3-D) mode. Continuous arterial blood sampling (0-3.5 minutes) was followed by discrete sampling (see eMethods 3 in the Supplement for the protocol). No significant group differences in injected mass, injected activity, or specific activity (>500 GBq/μmol) were noted (eTable 1 in the Supplement). High-resolution structural 3-D T1-weighted images were acquired on a PET/magnetic resonance hybrid 3-T scanner (Ingenuity; Philips) (see eMethods 4 in the Supplement for the sequences).

##### Study 2

Dynamic, continuous 90-minute PET scans were acquired after a bolus injection of [^11^C]MePPEP (mean [SD], 314 [34.4] MBq) using a scanner in 3-D mode (HiRez Biograph 6 CT44931; Siemens). Continuous arterial blood sampling (0-15 minutes) was followed by discrete sampling (see eMethods 3 in the Supplement for the protocol). No significant group differences in injected mass, injected activity, or specific activity were noted (eTable 1 in the Supplement). High-resolution structural 3-D T1-weighted images were acquired on a 3-T scanner (MR750; GE Healthcare) (see eMethods 4 in the Supplement for the sequences).

### Analysis

#### Imaging Analysis

Standard preprocessing pipelines were implemented for each study (see eMethods 5 in the Supplement for methods). Cannabinoid 1 receptor availability was indexed using the V_T_ of the respective tracer, calculated using the Logan graphical method with a metabolite-corrected arterial plasma input function^32^ (see eMethods 6 in the Supplement for model validation).

The anterior cingulate cortex,^33^ thalamus,^34^ hippocampus,^35^ and striatum^36^ were the primary regions of interest (ROIs), given evidence implicating them in the pathophysiology of schizophrenia and that CB1R regulates synaptic transmission in these ROIs.^37,38,39,40,41^ The ROIs were obtained from the Hammersmith atlas, a standard, probabilistic neuroanatomical atlas.^42^ To determine the influence of gray matter, ROI analyses were repeated when restricting the analysis to gray matter. Gray matter masks were obtained by binarizing segmented gray matter from T1-weighted images and applying this to the Hammersmith atlas.^42^

Cumulative movement and motion spikes were recorded (see eMethods 7 in the Supplement for movement parameters recorded). To determine whether volumetric group differences influenced our findings, primary ROI volumes were compared between groups using voxel-based morphometry (see eMethods 8 in the Supplement for methods). To enable comparison with previous studies, group differences in CB1R were investigated for additional ROIs defined using the Hammersmith atlas^42^ (see eMethods 9 in the Supplement for methods). A voxelwise analysis was conducted to investigate group differences in CB1R across the whole brain (see eMethods 10 in the Supplement).

#### Statistical Analysis

Data were analyzed from June 20, 2016, through February 12, 2019. SPSS, version 22 (IBM Corp), was used for statistical analyses. Data normality and sphericity were assessed using the Shapiro-Wilk test and the Mauchly test of sphericity, respectively. Categorical clinical, demographic, and experimental variables were assessed using χ^2^ tests; continuous variables were assessed using independent-sample *t* tests.

To determine whether CB1R availability was lower in patients, a repeated-measures analysis of variance using a 2 (group) × 4 (ROI) design was used for each study. Significant group × ROI interaction effects were explored using post hoc independent-sample *t* tests. To determine the influence of gray matter, this analysis of variance was repeated using gray matter–masked ROIs. Mean group differences were calculated for each ROI for each study, using the Hedges *g* effect size calculated as m1 minus m2 divided by the pooled, weighted SD, where m1 indicates mean CB1R for group 1 and m2, mean CB1R for group 2.

To determine whether our findings were influenced by potential confounding variables, a repeated-measures analysis of covariance using a 2 (group) × 4 (ROI) design included the quantity of current tobacco use (mean cigarettes per day) and lifetime cannabis exposure (mean number of joints) as covariates. Because group differences in cumulative movement occurred in study 1 but not study 2, a repeated-measures analysis of covariance using 2 (group) × 4 (ROI) design included movement as a covariate for study 1. To further evaluate whether potential confounds could influence CB1R availability, multiple linear regressions were conducted including tobacco use (current use, quantity of current use) or cannabis use (prior use, quantity of lifetime use) as independent variables and CB1R availability as the dependent variable. This analysis was performed separately for each ROI and for each study. To determine whether age was associated with CB1R availability, linear regressions including age as the independent variable and CB1R availability as the dependent variable were performed. These analyses were performed separately for each ROI and for each study.

Exploratory Pearson correlations investigated the association between the V_T_ of [^11^C]MePPEP for each ROI and (1) total Positive and Negative Syndrome Scale symptom severity and (2) cognitive functioning, as determined by Wechsler Digit Symbol Coding Test performance. The significance threshold was *P* < .05 (2 tailed) for all statistical tests. Bonferroni corrections for multiple comparisons were applied.

## Results

### Demographics and Experimental Variables

A total of 58 individuals participated (mean [SD] age of controls, 27.16 [5.93] years; mean [SD] age of patients, 26.96 [4.55] years). In study 1, 11 healthy male volunteers (mean [SD] age, 27.18 [5.86] years) were compared with 7 male patients with FEP (mean [SD] age, 26.80 [5.40] years); in study 2, 20 male healthy volunteers (mean [SD] age, 27.15 [6.12] years) were compared with 20 male patients with FEP (mean [SD] age, 27.00 [5.06] years). No significant group differences were found for age; ethnicity; use of alcohol, tobacco, or cannabis (Table 1 and Table 2); weight; body mass index; injected radiotracer dose; or injected mass in either study (eTable 1 in the Supplement). No significant group differences were found in tissue volumes of primary ROIs in either study (eResults 1 in the Supplement). In study 1, but not study 2, patients relative to controls showed significantly greater cumulative movement (mean [SD], 8.05 [3.36] vs 12.00 [3.68] mm; *P* = .03) (eTable 1 in the Supplement).

### CB1R Availability

#### Study 1

**Figure 1.**
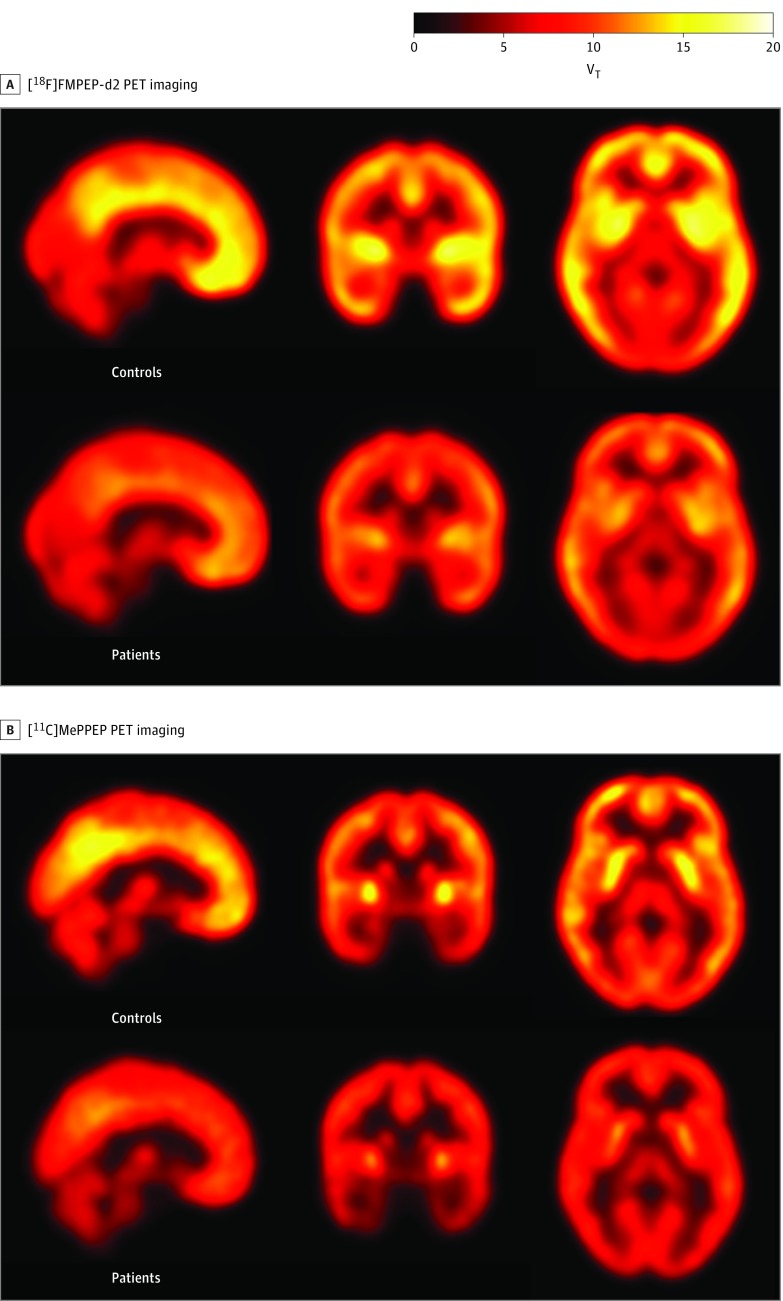
Cannabinoid 1 Receptor Availability Measured Using Positron Emission Tomographic (PET) Imaging Cannabinoid 1 receptor availability was significantly lower in patients with first-episode psychosis relative to healthy volunteers as determined by the distribution volume (V_T_; measured in milliliters per cubic centimeter) of radiotracers fluoride 18–labeled FMPEP-d_2_ (*F*_1,16_ = 19.84; *P* < .001) and carbon 11–labeled MePPEP (*F*_1,38_ = 4.96; *P* = .03). Images are mean parametric maps for controls (top row of A and B) and patients (bottom row of A and B) in each respective study. Brain regions with relatively higher distribution volumes of the respective radiotracer are shown in yellow.

**Figure 2.**
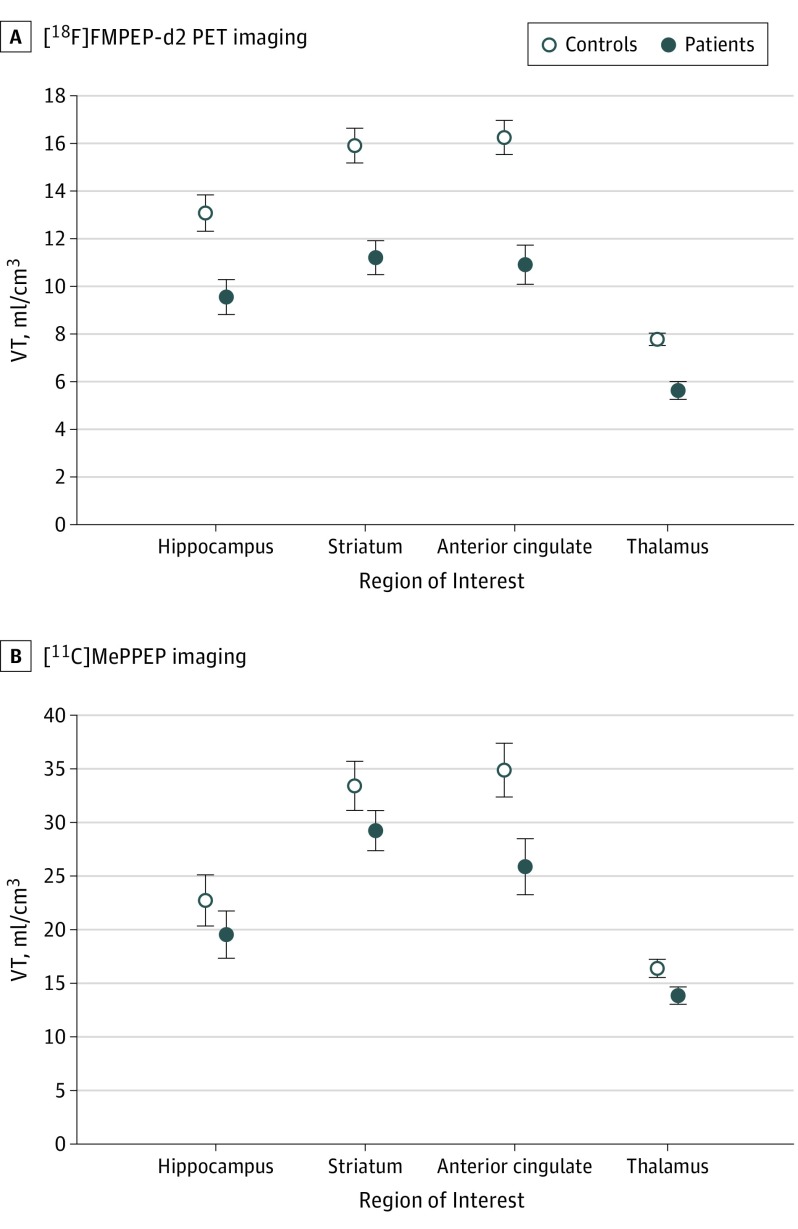
Cannabinoid 1 Receptor Availability Across Regions of Interest Cannabinoid 1 receptor availability measured by positron emission tomographic (PET) imaging was significantly lower in each region of interest in patients taking antipsychotic medication with first-episode psychosis relative to matched controls (panel A) and untreated patients with first-episode psychosis relative to matched controls (panel B). Data are expressed as mean (SD) of the distribution volume (V_T_) of fluoride 18–labeled FMPEP-d_2_ and carbon 11–labeled MePPEP radiotracers.

Data were normally distributed, and sphericity assumptions were met (χ^2^ = 4.67; *P* = .46). We found a significant main effect of group (*F*_1,16_ = 19.84; *P* < .001) and significant group × region interaction (*F*_3,48_ = 4.31; *P* = .01) (Figures 1 and 2 and eFigures 1-4 in the Supplement). Findings were unchanged when including cumulative movement as a covariate. Post hoc tests indicated that patients relative to controls showed significantly lower V_T_ in the anterior cingulate cortex (*t*_16_ = −4.48; *P* < .001; Hedges *g* = 1.2), hippocampus (*t*_16_ = −2.98; *P* = .006; Hedges *g* = 1.4), thalamus (*t*_16_ = −4.67; *P* < .001; Hedges *g* = 1.4), and striatum (*t*_16_ = −4.08; *P* = .001; Hedges *g* = 1.9).

#### Study 2

Data were normally distributed, but because sphericity assumptions were not met (χ^2^ = 14.74; *P* = .01), Greenhouse-Geisser estimates were used. We found a significant main effect of group (*F*_1,38_ = 4.96; *P* = .03) (Figures 1 and 2 and eFigures 5-8 in the Supplement), with V_T_ lower in the patients in the anterior cingulate cortex (Hedges *g* = 0.8), hippocampus (Hedges *g* = 0.5), striatum (Hedges *g* = 0.4), and thalamus (Hedges *g* = 0.7). Because the group × region interaction was not significant (*F*_2.33, 88.59_ = 1.11; *P* = .35), post hoc tests for individual ROIs were not conducted.

### CB1R Availability, Symptoms, and Cognition

**Figure 3.**
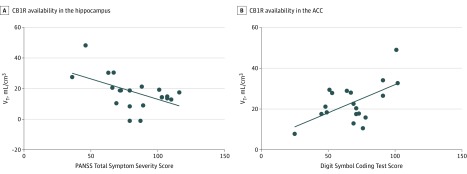
Association Between Cannabinoid 1 Receptor (CB1R) Availability and Symptom Severity and Cognitive Functioning Cannabinoid 1 receptor availability in the hippocampus and anterior cingulate cortex (ACC) was indexed using the distribution volume (V_T_) of carbon 11–labeled MePPEP. A, The distribution volume in the hippocampus was inversely associated with Positive and Negative Syndrome Scale total symptom severity scores (*R* = −0.50; *P* = .02). Scores ranged from 36 to 116, where higher scores indicated greater symptom severity. B, Distribution volume in the ACC was positively associated with cognitive functioning, as determined by the Digit Symbol Coding Test (*R* = 0.60; *P* = .01). Scores range from 25 to 102, where lower scores indicate greater cognitive impairment. Diagonal lines index the strength of a linear relationship between 2 variables, where a Pearson correlation coefficient of 1 indicates a positive association and −1 indicates a negative association.

We found a significant inverse association between Positive and Negative Syndrome Scale total symptom severity and hippocampal V_T_ of [^11^C]MePPEP (*R* = −0.50; *P* = .02) and a significant positive association between cognitive function, as determined by the Wechsler Digit Symbol Coding Test performance, and the V_T_ of [^11^C]MePPEP in the striatum (*R* = 0.50; *P* = .03) and anterior cingulate cortex (*R* = 0.60; *P* = .01) (Figure 3). The latter survived Bonferroni correction. No other significant associations were found.

### Additional Analyses

Findings for both studies were unchanged when restricting ROI analyses to gray matter or when including tobacco and cannabis use as covariates (eResults 2 and 3 in the Supplement). There were no significant differences in ROI volumes between groups in either study (eResults 1 in the Supplement). In study 1, patients relative to controls showed significantly lower V_T_ in additional ROIs (including frontal, parietal, temporal, and occipital lobes [eResults 1 in the Supplement]) but not in study 2 (eResults 4 in the Supplement). In voxelwise analyses, patients relative to controls showed lower V_T_ in temporal regions in studies 1 and 2; however, in study 1, patients relative to controls also showed lower V_T_ in frontal regions (eResults 5 in the Supplement). Tobacco use, cannabis use, and age were not significantly associated with CB1R availability (eTable 2 and eResults 6-9 in the Supplement).

## Discussion

Cannabinoid 1 receptor availability, as determined by 2 different CB1R-selective PET radiotracers, was significantly lower in untreated and antispychotic-treated patients with FEP relative to controls. Exploratory analyses indicated that lower CB1R levels were associated with greater symptom severity and poorer cognitive functioning.

Our findings are consistent with ex vivo literature showing that antipsychotic-treated patients have lower levels of CB1R messenger RNA and lower CB1R protein expression.^13,14,15,43^ However, they are inconsistent with an in vivo study reporting higher CB1R availability in FEP^20^ using [^18^F]MK-9470 without arterial blood sampling. Arterial blood sampling is necessary for full V_T_ quantification,^23^ and 2 in vivo studies have addressed this limitation by using arterial blood sampling in samples that included chronic and antipsychotic-treated patients. Although higher CB1R levels were found in the pons (N = 9) using [^11^C]OMAR,^21^ this finding did not survive correction for multiple comparisons, and a subsequent, larger study using [^11^C]OMAR^22^ reported lower CB1R availability in male patients (N = 25).

Our findings extend these previous results to show lower CB1R availability in FEP in 2 independent samples, including a sample of patients who were medication naive and free from all pharmacological treatments. Our finding that CB1R levels were lower in patient cohorts irrespective of antipsychotic medication use is consistent with preclinical literature indicating that antipsychotics do not alter CB1R density in rodents.^44^ Our finding that cortical CB1R availability is associated with poorer cognitive function is consistent with preclinical literature showing that CB1R agonists administered centrally to the medial prefrontal cortex impair cognition.^45,46,47^

### Interpretation and Implications for the Neurobiology of Psychosis

Because [^11^C]MePPEP and [^18^F]FMPEP-d_2_ are not displaced by methanandamide (AEA analogue),^48^ V_T_ is thought to primarily reflect receptor density. Although V_T_ is the sum of specific and nonspecific binding, both radiotracers have high levels of specific binding, ranging from 80% to 89%.^48,49^ Therefore, lower V_T_ in patients is likely to predominantly reflect specific CB1R binding.

Although the mechanism underlying lower CB1R in psychosis is unclear, exposure to synthetic CB1R agonists or an AEA analogue leads to CB1R internalization, wherein extracellular CB1R expression is decreased via endocytosis.^50,51^ Therefore, lower CB1R levels in patients may be due to CB1R internalization, secondary to the effects of higher endogenous AEA levels in patients with FEP who do not use cannabis.^11^ In line with this possibility, AEA administration in mice that are deficient in an enzyme involved in AEA degradation (fatty acid amide hydrolase) show region-specific reductions in CB1R levels.^52^ However, because no studies have tested this in humans, this interpretation is speculative.

Although what might precipitate high AEA levels in patients who do not use cannabis is unclear,^11^ stress exposure, a key risk factor for schizophrenia,^53^ increases AEA levels^54^ and decreases CB1R density in the hippocampus.^55^ After illness onset, the experience of hallucinations and delusions may also increase stress levels^56^ and, in turn, the production of AEA,^54^ exacerbating reductions in CB1R levels.^55^ However, studies are needed to investigate whether stress may precipitate AEA and CB1R alterations in humans. Alternatively, lower CB1R levels may be secondary to reductions in synaptic density in the hippocampus and frontal cortices seen in schizophrenia.^57^

Because CB1R binding inhibits calcium entry into the presynaptic neuron via N-, P-, and Q-type calcium channels,^58,59^ the presence of fewer CB1Rs may dysregulate calcium and potassium channels, leading to neurochemical alterations in psychosis.^34,35,36,60,61^ Because CB1Rs modulate neurotransmitters implicated in psychosis, including dopamine,^62^ glutamate,^63^ and γ-aminobutyric acid,^58^ future studies are needed to investigate whether CB1R alterations precipitate other neurochemical alterations in psychosis.

Although CB1R antagonists are not licensed owing to adverse effects,^64^ they have been found to reduce deficits induced by a phencyclidine model of psychosis.^65^ However, CB1R-negative allosteric modulators (eg, cannabidiol) are associated with few adverse effects^66^ and have been shown to reduce CB1R agonist efficacy and potency, preventing CB1R internalization.^67^ Taken together, these results support further work to explore the therapeutic potential of CB1R modulators in schizophrenia.

### Strengths and Limitations

Because studies 1 and 2 were analyzed separately, a strength of this work was that we reported consistent findings despite using different samples and radiotracers, indicating that findings generalize across methods. A limitation of study 1 was that we were unable to record plasma free fraction or specific activity, owing to high specific activity (>500 GBq/μmol) for [^18^F]FMPEP-d_2_. Moreover, we were unable to investigate associations between CB1R availability and symptoms or cognition owing to limited power and because cognition was not measured in study 1. Because a genetic variant of the cannabinoid 1 receptor gene (*CNR1*) is associated with altered [^18^F]FMPEP-d_2_ V_T_,^68^ genetic differences may contribute to study 1 findings.

In study 2, the association between cortical CB1R availability and cognition survived multiple comparisons corrections, but the association between CB1R availability and symptom severity did not. Moreover, causal inferences regarding the role of CB1R in the etiology of psychosis cannot be made, given our cross-sectional design. Although the Wechsler Digit Symbol Coding Test is highly correlated with global cognitive impairments,^31^ this measure lacks specificity at the expense of sensitivity, and which precise aspect of cognition is affected is unclear.^69^ This measure was chosen to minimize participant burden but, given our findings, determining whether CB1R availability is associated with specific aspects of cognition would be useful.

Although the test-retest variability of V_T_ estimates is comparable for [^18^F]FMPEP-d_2_ and [^11^C]MePPEP,^48^ the V_T_ variance estimates were smaller for [^18^F]FMPEP-d_2_ than [^11^C]MePPEP, which may explain why effect sizes were larger in study 1. Although a significant group × region interaction occurred in study 1 but not 2, post hoc tests indicated that CB1R levels were lower in all regions in study 1, consistent with study 2. Notwithstanding this, the interaction indicated that group differences were larger in specific regions. This finding may reflect the inclusion of patients with affective psychoses in study 1 but not study 2. Patients showed greater cumulative movement in study 1 but not 2. Nevertheless, findings from study 1 remained unchanged when including cumulative movement as a covariate. We did not observe volumetric group differences in any region of interest, indicating that partial volume effects are unlikely to be a major confound.

A strength of both studies was that volunteers with cannabis abuse or dependence or positive results on urine toxicology tests detecting cannabis and other illicit substances before scanning were excluded. However, because individuals using cannabis occasionally may have 11-nor-9-carboxy-Δ9-tetrahydrocannnabinol concentrations below the limit of sensitivity (50 ng/mL),^70^ infrequent cannabis use may have been undetected. Although some volunteers had previously used cannabis, 1 month of abstinence has been shown to normalize CB1R levels.^71^ Because findings for both studies remained unchanged when including prior cannabis use or lifetime cannabis use in our model, cannabis use is unlikely to be a significant confound. Although only men were included owing to sex differences in CB1R,^24^ future studies are needed to determine whether female patients show CB1R alterations.

## Conclusions

Cannabinoid 1 receptor availability is lower in male patients with FEP, and this is associated with poorer cognitive function and greater symptom severity. These findings indicate that CB1R alterations may contribute to the pathophysiology of psychosis.

## Notes

Supplement. **eMethods 1.** Full Exclusion Criteria **eMethods 2.** Measures for Tobacco, Alcohol, and Cannabis Use **eMethods 3.** PET Imaging Acquisition Parameters **eMethods 4.** MRI Imaging Acquisition Parameters **eMethods 5.** PET Image Analysis: Preprocessing Methods **eMethods 6.** CB1R Availability: Kinetic Modeling Validation **eMethods 7.** Movement Parameters **eMethods 8.** Volumetric Image Analysis **eMethods 9.** Cannabinoid Receptor Availability: Additional Analyses **eMethods 10.** Voxelwise Analysis: Additional Analyses **eTable 1.** Experimental Parameters for Study 1 and 2 **eTable 2.** Cannabinoid 1 Receptor Availability in Healthy Volunteers and First Episode Psychosis Patients Using a Whole Brain Voxelwise Analysis for Study 1 and 2 **eResults 1.** Volumetric Image Analysis **eResults 2.** Cannabinoid Receptor Availability: Gray Matter Masking **eResults 3.** Cannabinoid Receptor Availability Controlling for Potential Confounders **eResults 4.** Cannabinoid Receptor Availability: Additional Region-of-Interest Analysis **eResults 5.** Cannabinoid Receptor Availability: Voxelwise Analysis **eResults 6.** Association Between CB1R Availability and Tobacco Use **eResults 7.** Association Between CB1R Availability and Cannabis Use **eResults 8.** Association Between CB1R Availability and Age **eResults 9.** Radiotracer Spatial Covariance **eFigure 1.** CB1R Availability in the Hippocampus in Patients Relative to Healthy Volunteers in Study 1 **eFigure 2.** CB1R Availability in the Striatum In Patients Relative to Healthy Volunteers in Study 1 **eFigure 3.** CB1R Availability in the Anterior Cingulate Cortex in Patients Relative to Healthy Volunteers in Study 1 **eFigure 4.** CB1R Availability in the Thalamus in Patients Relative to Healthy Volunteers in Study 1 **eFigure 5.** CB1R Availability in the Hippocampus in Patients Relative to Healthy Volunteers in Study 2 **eFigure 6.** CB1R Availability in the Striatum in Patients Relative to Healthy Volunteers in Study 2 **eFigure 7.** CB1R Availability in the Anterior Cingulate Cortex in Patients Relative to Healthy Volunteers in Study 2 **eFigure 8.** CB1R Availability in the thalamus in Patients Relative to Healthy Volunteers in Study 2 **eReferences.**
