# Supplementary material for: In Vivo Availability of Cannabinoid 1 Receptor Levels in Patients With First-Episode Psychosis
Source: JAMA Psychiatry. 2019 Jul 3;76(10):1074–84. doi: 10.1001/jamapsychiatry.2019.1427 (PMC6613300; doi:10.1001/jamapsychiatry.2019.1427)
Supplement: Supplement. — eMethods 1. Full Exclusion Criteria eMethods 2. Measures for Tobacco, Alcohol, and Cannabis Use eMethods 3. PET Imaging Acquisition Parameters eMethods 4. MRI Imaging Acquisition Parameters eMethods 5. PET Image Analysis: Preprocessing Methods eMethods 6. CB1R Availability: Kinetic Modeling Validation eMethods 7. Movement Parameters eMethods 8. Volumetric Image Analysis eMethods 9. Cannabinoid Receptor Availability: Additional Analyses eMethods 10. Voxelwise Analysis: Additional Analyses eTable 1. Experimental Parameters for Study 1 and 2 eTable 2. Cannabinoid 1 Receptor Availability in Healthy Volunteers and First Episode Psychosis Patients Using a Whole Brain Voxelwise Analysis for Study 1 and 2 eResults 1. Volumetric Image Analysis eResults 2. Cannabinoid Receptor Availability: Gray Matter Masking eResults 3. Cannabinoid Receptor Availability Controlling for Potential Confounders eResults 4. Cannabinoid Receptor Availability: Additional Region-of-Interest Analysis eResults 5. Cannabinoid Receptor Availability: Voxelwise Analysis eResults 6. Association Between CB1R Availability and Tobacco Use eResults 7. Association Between CB1R Availability and Cannabis Use eResults 8. Association Between CB1R Availability and Age eResults 9. Radiotracer Spatial Covariance eFigure 1. CB1R Availability in the Hippocampus in Patients Relative to Healthy Volunteers in Study 1 eFigure 2. CB1R Availability in the Striatum In Patients Relative to Healthy Volunteers in Study 1 eFigure 3. CB1R Availability in the Anterior Cingulate Cortex in Patients Relative to Healthy Volunteers in Study 1 eFigure 4. CB1R Availability in the Thalamus in Patients Relative to Healthy Volunteers in Study 1 eFigure 5. CB1R Availability in the Hippocampus in Patients Relative to Healthy Volunteers in Study 2 eFigure 6. CB1R Availability in the Striatum in Patients Relative to Healthy Volunteers in Study 2 eFigure 7. CB1R Availability in the Anterior Cingulate Cortex in Patients Relative to Healthy Volunte [file jamapsychiatry-76-1074-s001.pdf]

Supplementary Online Content

Borgan F, Laurikainen H, Veronese M, et al; METSY Group. In vivo availability of cannabinoid 1 receptor levels in patients with first-episode psychosis. *JAMA Psychiatry.* Published online July 3, 2019. doi:10.1001/jamapsychiatry.2019.1427

eMethods 1. Full Exclusion Criteria

eMethods 2. Measures for Tobacco, Alcohol, and Cannabis Use

eMethods 3. PET Imaging Acquisition Parameters

eMethods 4. MRI Imaging Acquisition Parameters

eMethods 5. PET Image Analysis: Preprocessing Methods

eMethods 6. CB1R Availability: Kinetic Modelling Validation

eMethods 7. Movement Parameters

eMethods 8. Volumetric Image Analysis

eMethods 9. Cannabinoid Receptor Availability: Additional Analyses

eMethods 10. Voxelwise Analysis: Additional Analyses

eTable 1. Experimental Parameters for Study 1 and 2

eTable 2. Cannabinoid 1 Receptor Availability in Healthy Volunteers and First Episode Psychosis Patients Using a Whole Brain Voxelwise Analysis for Study 1 and 2

eResults 1. Volumetric Image Analysis

eResults 2. Cannabinoid Receptor Availability: Gray Matter Masking

eResults 3. Cannabinoid Receptor Availability Controlling for Potential Confounders

eResults 4. Cannabinoid Receptor Availability: Additional Region-of-Interest Analysis

© 2019 Borgan F et al. *JAMA Psychiatry.*

eResults 5. Cannabinoid Receptor Availability: Voxelwise Analysis
eResults 6. Association Between CB1R Availability and Tobacco Use
eResults 7. Association Between CB1R Availability and Cannabis Use
eResults 8. Association Between CB1R Availability and Age
eResults 9. Radiotracer Spatial Covariance
eFigure 1. CB1R Availability in the Hippocampus in Patients Relative to Healthy Volunteers in Study 1
eFigure 2. CB1R Availability in the Striatum In Patients Relative to Healthy Volunteers in Study 1
eFigure 3. CB1R Availability in the Anterior Cingulate Cortex in Patients Relative to Healthy Volunteers in Study 1
eFigure 4. CB1R Availability in the Thalamus in Patients Relative to Healthy Volunteers in Study 1
eFigure 5. CB1R Availability in the Hippocampus in Patients Relative to Healthy Volunteers in Study 2
eFigure 6. CB1R Availability in the Striatum in Patients Relative to Healthy Volunteers in Study 2
eFigure 7. CB1R Availability in the Anterior Cingulate Cortex in Patients Relative to Healthy Volunteers in Study 2
eFigure 8. CB1R Availability in the thalamus in Patients Relative to Healthy Volunteers in Study 2
eReferences.

This supplementary material has been provided by the authors to give readers additional information about their work.

© 2019 Borgan F et al. *JAMA Psychiatry.*

eMethods 1. Full Exclusion Criteria

Study 1

Exclusion criteria for all volunteers were as follows: 1) ages <18 or >60; 2) a history of a head injury leading to loss of consciousness, 3) personal or family history of neurological, neurodevelopmental, endocrine or cardiovascular health problems, 4) contraindications to MRI safety, 5) current or lifetime history of substance use or dependence as determined by the Structured Clinical Interview for DSM-IV-TR (SCID-I/P), 6) current or recent (within last month) recreational use of illicit substances, 7) screened positive on a THC urine toxicology test that can detect THC metabolite THCCOOH for up to 30 days (InstAlert; 50 ng/ml cut off), or 8) screened positive on a multi-panel urine drug screen (InstAlert) detecting the following substances: amphetamine (500ng/ml cut-off), buprenorfine (5ng/ml cut-off), cocaine (300ng/ml cut-off), methadone (300ng/ml cut-off), opiates (300ng/ml cut-off).

Study 2

Exclusion criteria for all volunteers were as follows: 1) ages <18 or >60; 2) history of a head injury leading to loss of consciousness; 3) personal or family history of neurological, neurodevelopmental, endocrine or cardiovascular health problems; 4) contraindications to MRI including the presence of metal plates, pins, bridges or dentures and pregnancy; 5) current or lifetime history of substance use or dependence as determined by the Structured Clinical Interview for DSM-IV-TR (SCID-I/P); 6) current or recent (within last month) recreational use of illicit substances; 7) screened positive on a THC urine toxicology test that can detect THC metabolite THCCOOH for up to 30 days (SureScreen, Diagnostics; 50 ng/ml cut off); 8) screened positive on a multi-panel drug screen detecting the following substances: amphetamine (300 ng/ml cut off), cocaine (150 ng/ml cut off), ketamine (1000 ng/ml cut off), marijuana (50 ng/ml cut off), methamphetamine (300 ng/ml cut off), opiates (2000 ng/ml cut off) (SureScreen Diagnostics).

eMethods 2. Measures for Tobacco, Alcohol, and Cannabis Use

Study 1

Alcohol use was recorded using the Audit questionnaire [1], tobacco use was recorded using a questionnaire adapted from the World Health Organization [2] and cannabis use was measured using an illicit substance use questionnaire described previously [3].

Study 2

Current and previous use of alcohol, nicotine and illicit substances were recorded. If volunteers reported current use, the quantity (cigarettes/alcohol units etc.), duration (years) and frequency (number of uses per week) of use was recorded. Prior cannabis use was recorded using the Cannabis Experiences questionnaire [4] as well the following variables: lifetime use (yes/no), age of first use (years), quantity of lifetime use (joints) and time since last use (days). Current tobacco use was defined as tobacco use on at least one occasion within the last week.

eMethods 3. PET Imaging Acquisition Parameters

Study 1

Non-continuous 120-minute PET scans were acquired using a brain dedicated PET (Siemens ECAT HRRT) in three-dimensional mode, after a bolus injection of 201 ± 11.10 MBq of [^{18}F]FMPEP-d_2, synthesized using methods described previously [5]. Subjects underwent the scan between 0-60 and 90-120 minutes. Two attenuation correction scans were acquired using a Cs^{137} point source before bolus injection and after 120 minutes to avoid attenuation correction bias induced by repositioning after the scan break. Emission data were reconstructed using a 3D-OSEM algorithm into 19 frames of increasing length (3x1 min, 5x3 min, 7x6 min and 4x7.5 min) with a 1.22x1.22x1.22 mm^3 isometric voxel-size. Continuous arterial blood sampling took place for the first 3.5 minutes of the

© 2019 Borgan F et al. *JAMA Psychiatry.*

scan. This was followed by discrete blood sampling at 4.5, 7.5, 11, 15, 20, 25, 30, 35, 40, 45, 50, 60, 70, 80, 100 minutes to measure plasma tracer radioactivity, and at 4.5, 11, 15, 30, 45, 60, 70, 80, 100 minutes to measure the fraction of unmetabolized radiotracer.

Study 2

Continuous 90-minute positron emission tomography (PET) scans were acquired on a PET/CT (Hi-Rez Biograph 6 CT44931) in three-dimensional mode, after a bolus injection of 314 ± 34.4 MBq of $[^{11}C]$MePPEP, synthesized using methods reported elsewhere [6,7]. CT scans were acquired prior to each PET scan for correction for attenuation and scatter. Continuous arterial blood sampling took place for the first 15 minutes of the scan which was followed by discrete blood sampling at 2, 5, 10, 15, 20, 25, 35, 40, 50, 60, 70, 80 and 90 minutes after the radioligand injection. Images were reconstructed with filtered back projection including corrections for attenuation and scatter.

eMethods 4. MRI Imaging Acquisition Parameters

Study 1

High-resolution 3D T1-weighted structural MRI images were acquired for all subjects on a Philips 3T Ingenuity PET/MR hybrid scanner using multi-shot turbo field echo and a 32 channel head coil (176 slices with isometric $1mm^3$ voxel size, in-plane matrix size of 256 x 256, FOV=176mm, TR=8.1; TE=3.7; TI=1s; flip angle=7°; slice thickness=1mm; slice gap=0mm).

Study 2

High-resolution 3D T1-weighted structural MRI images were acquired for anatomical co-localisation on a General Electric MR750 3.0T scanner (in-plane matrix size of 256 × 256, FOV = 26.0 mm) using whole-brain, interleaved bottom-up acquisition, sagittal orientation and an 8 channel head coil (TR = 7.34 mm, TE = 3.036 mm, inversion time = 4 seconds, flip angle = 11°, slice thickness = 1.2 mm, slice gap = 1.2 mm).

eMethods 5. PET Image Analysis: Preprocessing Methods

Study 1

Data pre-processing was performed using a combination of Statistical Parametric Mapping 12 (http://www.fil.ion.ucl.ac.uk/spm) and Matlab R2014b (The Mathworks Inc., Sherborn, Massachusetts). Motion correction was applied for all PET scans as follows. Attenuation and scatter corrected images were realigned to a single reference frame (the 12th frame, which contained the highest uptake average). Realigned frames were then summated to create single-subject motion-corrected maps which were then used for MRI and PET co-registration, prior to PET data quantification. T1-weighted structural images were co-registered to the PET image using rigid body transformations. Normalization parameters were obtained by warping the co-registered structural MRI to MNI space (International Consortium for Brain Mapping ICBM/MNI) using probabilistic tissue classification with bias correction. The inverse of these parameters was used to fit a neuroanatomical atlas to each individual PET scan using the Hammersmith atlas (Hammers et. al 2003).

Decay corrected whole blood tissue activity curves (TAC) derived from automatic blood pump sampling were converted to plasma activity using hematocrit and a population derived tracer specific distribution function. Automated and manual sample TACs were then combined and PET count rate curves were used to reference peak tissue activity to correct for temporal delay between blood sample measurement and target the tissue data. Plasma and whole blood TAC values were extrapolated from 100 to 120 minutes using a biexponential function fit starting at two times the peak activity location. The time delay of the peak radioactivity reaching tissue and blood samples was corrected using PET count rate curves to reference peak tissue activity. The resulting whole blood and plasma TACs were corrected for the fraction of unchanged tracer, which was measured from arterial plasma using thin-layer chromatography and interpolated

© 2019 Borgan F et al. *JAMA Psychiatry.*

with an extended version of Hill model fit to the measured un-metabolized fraction time series. The resulting plasma activity concentration curve, corrected for metabolites, was used as parent input for modelling.

Study 2

Data pre-processing was performed using a combination of Statistical Parametric Mapping 12 (http://www.fil.ion.ucl.ac.uk/spm) and FSL (http://www.fsl.fmrib.ox.ac.uk/fsl) functions, as implemented in MIAKAT (miakat.org). Motion correction was applied to non-attenuation corrected images [8]. Non-attenuated corrected frames were realigned to a single "reference" frame (corresponding to that with the highest number of counts) by employing a mutual information algorithm. The transformation parameters were then applied to the corresponding attenuated-corrected dynamic images, creating a movement-corrected dynamic image which was used for the analysis. Realigned frames were then summated to create single-subject motion-corrected maps which were then used for MRI and PET co-registration, prior to PET data quantification. T1-weighted structural images were co-registered to the PET image using rigid body transformation. Normalization parameters were obtained by warping the co-registered structural MRI to MNI space (International Consortium for Brain Mapping ICBM/MNI) using bias-corrected segmentation. The inverse of these parameters was used to fit a neuroanatomical atlas to each individual PET scan using the Hammersmith atlas (Hammers et. al 2003). Whole blood time-activity curves (TACs) were fitted using a multi-exponential function as derived by Feng's model (20). For each scan, a time delay was fitted and applied to the input functions (both parent and whole blood TACs) to account for any temporal delay between blood sample measurement and target the tissue data.

eMethods 6. CB1R Availability: Kinetic Modelling Validation

Study 1 & 2

While the use of Logan has been previously validated for FMPEP, returning test-retest data comparable to two-tissue compartmental modelling [9] we validated the use of Logan by comparing V_T estimates with coefficient variation <10% derived from Logan and 2TCM modelling. In this context, we demonstrated that the mean relative difference between Logan and 2TCM was low (whole brain mrd: -4%+/-11%) and that V_T estimates derived using these models were highly correlated (Pearson's correlation from 0.7 to 0.94, mean+/-sd: 0.83+/-0.13) in both controls and patients. Logan led to lower between-subject variability compared to 2TCM (average across region 38% for Logan vs 74% for 2TCM) which is consistent with the literature and the performance obtained by the best method proposed by Barros and colleagues [7]. In view of this, and to be consistent across both studies, we used the Logan approach for our analyses.

eMethods 7. Movement Parameters

Study 1 & 2

For studies 1-2, cumulative scanner movement was defined as the sum of total frame-to-frame movement during imaging acquisition. For studies 1-2, motion spikes were defined as frame-to-frame scanner movement exceeding 5mm since the resolution of PET images were 5mm.

eMethods 8. Volumetric Image Analysis

Study 1 & 2

A voxel-based morphometry (VBM) analysis was conducted using SPM12 in order to determine if there were volumetric differences between patients and controls. Structural T1-weighted structural scans were segmented, warped to a template created using the DARTEL algorithm which improves the accuracy of inter-subject registration and realignment, normalised into MNI space and smoothed using an 8mm Gaussian kernel. To identify if there were volumetric differences between patients and controls, global tissues volumes were compared using independent samples t-test was conducted in SPM12 including age

© 2019 Borgan F et al. *JAMA Psychiatry.*

and total intracranial volume as covariates. Tissue volumes were compared between patients and controls in whole-brain and region of interest analyses of the anterior cingulate, thalamus, hippocampus and striatum. The height threshold was set to p=0.001 and peak-level family-wise error corrected thresholds (p<0.05) were used.

eMethods 9. Cannabinoid Receptor Availability: Additional Analyses

Study 1 & 2

To determine if CB1R availability was lower in patients, a repeated measures ANOVA using a 2 (group: control vs. patient) x 13 (region: amygdala, caudate, putamen, insula, cerebellum, posterior cingulate, temporal lobe, parietal lobe, occipital lobe, frontal lobe) design was used for each dataset. These regions of interest were also defined using the Hammersmith atlas [10]. The main effect of group tested whether the V_T of the respective tracer was different between patients and controls, and the group x region interaction tested whether mean V_T across ROIs were different between groups, where a null result indicates a global reduction in V_T. Interaction effects were explored with post-hoc independent sample t-tests (two-tailed).

eMethods 10. Voxelwise Analysis: Additional Analyses

Study 1 & 2

An exploratory voxel-wise analysis was also conducted using SPM12 in order to determine if there were whole-brain voxel-wise differences in CB1R availability between patients and controls. The height threshold was set to p=0.001 and peak-level family-wise error corrected thresholds (p<0.05) were used.

© 2019 Borgan F et al. *JAMA Psychiatry.*

eTable 1. Experimental Parameters for Study 1 and 2

Study 1: ([^{18}F]FMPEP-d_2) - medicated

	Healthy volunteers	FEP patients	t	df	p
N	11	7			
Weight (kg)	M=81.91; SD=9.66	M=94.00; SD=19.83	1.743	16	0.10
Body mass index	M=25.28; SD=3.74	M=29.35; SD=6.62	1.681	16	0.11
Dose (Mbq)	M=199.64; SD=13.00	M=203.29; SD=8.82	0.650	16	0.53
Specific activity	>500 Gbq/micromole	>500 Gbq/micromole	NA	NA	NA
Injected mass (ng)	M<189.05; SD=12.31	M=<192.51; SD=8.36	0.650	16	0.53
Total scanner motion (mm)*	M=8.05; SD=3.36	M=12.00; SD=3.68	2.350	16	0.03

Study 2: ([^{11}C]MePPEP) – un-medicated

	Healthy volunteers	FEP patients	t	df	p
N	20	20			
Weight (kg)	M=78.29; SD=13.26	M=85.09; SD=14.17	-1.568	38	0.13
Body mass index	M=25.47; SD=3.78	M=26.65; SD=5.24	-0.674	26	0.51
Dose (MBq)	M=311.32; SD=44.87	M=311.50; SD=27.48	0.016	38	0.98
Injected mass (µg)	M=4.31; SD=1.60	M=4.72. SD=2.46	-0.583	38	0.56
Specific activity GBq/µmol	M=97.32; SD=287.91	M=158.38; SD=556.02	-.436	38	0.67
Fp (% if >1 or fraction if <1)	M=0.19; SD=4.68	M=0.16; SD=0.05	1.758	38	0.09
Total scanner motion (mm)*	M=12.58; SD=4.68	M=13.78; SD=5.95	-0.709	38	0.48

*Total scanner motion was defined as the sum of total frame-to-frame movement during imaging acquisition. Experimental parameters for the positron emission tomography scans for study 1 and 2 including weight, body mass index, dose, specific activity, injected mass, total scanner motion. Body mass index, calculated using methods described previously [11]. FEP=first episode psychosis; N=number; kg=kilograms; mm=millimetres; MBq=megabecquerel; µg=microgram; umol=micromoles; GBq=gigabecquerel.

© 2019 Borgan F et al. *JAMA Psychiatry.*

eResults 1. Volumetric Image Analysis

To identify if there were volumetric differences between patients and controls, tissue volumes were compared between patients and controls in whole-brain and region of interest analyses of the anterior cingulate, thalamus, hippocampus and striatum.

Study 1

There were no significant group differences in tissue volumes between patients and controls in whole-brain and region of interest analyses of the anterior cingulate, thalamus, hippocampus and striatum. No statistics are reported because there were no suprathreshold clusters.

Study 2

There were no significant group differences in tissue volumes between patients and controls in whole-brain and region of interest analyses of the anterior cingulate, thalamus, hippocampus and striatum. No statistics are reported because there were no suprathreshold clusters.

eResults 2. Cannabinoid Receptor Availability: Gray Matter Masking

To determine if differences in gray matter volumes in ROIs influenced our results, the analyses were repeated using a gray matter mask applied to each ROI to restrict the image analysis to gray matter. The results for each study are as follows:

Study 1

Data were normally distributed and assumptions of sphericity were not violated, x^2=4.67, p=0.46. There was a significant main effect of group on V_T (ml/cm^3) of [^{18}F] FMPEP-d_2 , F(1,16)=19.981, p<0.001 in the anterior cingulate cortex (Hedge's g=2.1), hippocampus (Hedge's g=1.5), striatum (Hedge's g=2.1) and thalamus (Hedge's g=2.2). There was also a significant main effect of region, F(1, 16)=98.99, p<0.001. There was also a significant group x region interaction, F(1,16)=4.05, p=0.012.

Study 2

Data were normally distributed and since assumptions of sphericity were violated, x^2=12.14, p=0.033, Greenhouse-Geisser estimates were used. There was a significant main effect of group on V_T (ml/cm^3) of [^{11}C]MePPEP, F(1,38)=5.736, p=0.022 in the anterior cingulate cortex (Hedge's g=0.8), hippocampus (Hedge's g=0.5), striatum (Hedge's g=0.4) and thalamus (Hedge's g=0.7). There was also a significant main effect of region, F(2.31, 88.59)=46.132, p<0.001. However, the group x region interaction effect was not significant, F(2.33, 88.59)=1.112, p=0.347.

eResults 3. Cannabinoid Receptor Availability Controlling for Potential Confounders

To determine if tobacco or lifetime cannabis use influenced our results, the analyses were repeated including data on these variables as covariates.

Study 1

Data were normally distributed and since assumptions of sphericity were not violated, x^2=5.05, p=0.41. There was a significant main effect of group on V_T (F(1,25)=6.47, p=0.01) . There was a significant interaction effect between group x region (F(3,42)=4.71, p=0.01). There were no significant interaction effects between region x the quantity of current tobacco use (F(3,42)=0.56, p=0.64) or between region x quantity of lifetime cannabis use (F(3,42)=0.74, p=0.53).

© 2019 Borgan F et al. *JAMA Psychiatry.*

Study 2

Data were normally distributed and since assumptions of sphericity were not violated, x^2=10.82, p=0.055. There was a significant main effect of group on V_T (F(1,25)=6.47, p=0.01) . There were no significant interaction effects between group x region (F(1, 25)=2.95, p=0.10), region x quantity of lifetime cannabis use (F(3,75)=2.53, p=0.06) or region x quantity of current tobacco use (F(3,75)=0.21, p=0.89.

eResults 4. Cannabinoid Receptor Availability: Additional Region-of-Interest Analysis

To determine if CB1R availability was lower in patients across additional brain regions for comparison with other studies, a repeated measures ANOVA using a 2 (group: control vs. patient) x 13 (region: amygdala, caudate, putamen, insula, cerebellum, posterior cingulate, temporal lobe, parietal lobe, occipital lobe, frontal lobe) design was used for each dataset. These regions of interest were also defined using the Hammersmith atlas [10]. The main effect of group tested whether the V_T of the respective tracer was different between patients and controls, and the group x region interaction tested whether mean V_T across ROIs were different between groups, where a null result indicates a global reduction in V_T. Interaction effects were explored with post-hoc independent sample t-tests (two-tailed).

Study 1

Data were normally distributed and since assumptions of sphericity were violated, x^2=165.143, p<0.001, Greenhouse-Geisser estimates were used. There was a significant main effect of group on V_T (F(1,16)=17.112, p=0.001. There was also a significant main effect of region on V_T (F(2.28, 36.49)=36.33, p<0.001. However, the group x region interaction did not reach statistical significance (F(2.28, 36.49)=2.86, p=0.06).

Study 2

Data were normally distributed and since assumptions of sphericity were violated, x^2=254.29, p<0.001, Greenhouse-Geisser estimates were used. There was no significant main effect of group on V_T (F(1, 38)=2.41, p=0.13. However, there was a significant main effect of region F(3.65, 138.58)=74.06, p<0.001. However, the group x interaction was not significant F(3.65, 138.58)=1.77, p=0.15.

eResults 5. Cannabinoid Receptor Availability: Voxelwise Analysis

Study 1

In a whole-brain voxel-wise analysis, patients relative to controls showed significantly lower V_T of [^{18}F]FMPEP-d_2 in a cluster encompassing the anterior and middle cingulate, superior and middle frontal gyrus, inferior orbital gyrus, middle and inferior temporal gyrus, inferior opercular gyrus, inferior frontal pars triangularis (see supplementary figure 1). There were no differences in the control<patient contrast.

Study 2

Patients relative to controls showed significantly lower V_T of [^{11}C]MePPEP in a cluster encompassing the inferior temporal gyrus and fusiform gyrus (T=4.98, pFWE<0.001) (see supplementary figure 2). There were no differences in the control<patient contrast.

eResults 6. Association Between CB1R Availability and Tobacco Use

Study 1

In a multiple linear regression, V_T of [^{18}F] FMPEP-d_2 in the thalamus was not significantly predicted by current tobacco use (β=-2.56, p=0.45) or the quantity of

© 2019 Borgan F et al. *JAMA Psychiatry.*

current tobacco use (cigarettes per day) (β=0.32, p=0.63) with an R^2=0.11. Similarly, the V_T of [^{18}F] FMPEP-d_2 in the hippocampus was not significantly predicted by current tobacco use (β=-1.14, p=0.87) or the quantity of current tobacco use (cigarettes per day) (β=-0.13, p=0.93) with an R^2=0.06. In line with this, the V_T of [^{18}F] FMPEP-d_2 in the striatum was not significantly predicted by current tobacco use (β=-4.15, p=0.60) or the quantity of current tobacco use (cigarettes per day) (β=0.52, p=0.74) with an R^2=0.05. Moreover, the V_T of [^{18}F] FMPEP-d_2 in the anterior cingulate was also not significantly predicted by current tobacco use (β=-5.78, p=0.50) 2.56 or the quantity of current tobacco use (cigarettes per day) (β=0.92, p=0.60) with an R^2=0.05.

Study 2

In a multiple linear regression, V_T of [^{11}C]MePPEP in the thalamus was not significantly predicted by current tobacco use (β=0.89, p=0.53) or the quantity of current tobacco use (cigarettes per day) (β=-0.15, p=0.63) with an R^2=0.04. The V_T of [^{11}C]MePPEP in the hippocampus was not significantly predicted by current tobacco use (β=1.51, p=0.71) or the quantity of current tobacco use (cigarettes per day) (β=-0.38, p=0.66) with an R^2=0.02. The V_T of [^{11}C]MePPEP in the striatum was not significantly predicted by current tobacco use (β=0.08, p=0.98) or the quantity of current tobacco use (cigarettes per day) (β=-0.31, p=0.68) with an R^2=0.007. The V_T of [^{11}C]MePPEP in the anterior cingulate cortex was not significantly predicted by current tobacco use (β=-0.69, p=0.86) or the quantity of current tobacco use (cigarettes per day) (β=-0.31, p=0.70) with an R^2=0.004.

eResults 7. Association Between CB1R Availability and Cannabis Use

Study 1

In a multiple linear regression, V_T of [^{18}F] FMPEP-d_2 in the thalamus was not significantly predicted by prior cannabis use (β=1.55, p=0.14) or the quantity of prior cannabis use (joints) (β=-0.31, p=0.46) with an R^2=0.16. Similarly, the V_T of [^{18}F] FMPEP-d_2 in the anterior cingulate was not significantly predicted by prior cannabis use (β=4.35, p=0.09) or the quantity of prior cannabis use (joints) (β=-0.66, p=0.52) with an R^2=0.22. In line with this, the V_T of [^{18}F] FMPEP-d_2 in the hippocampus not significantly predicted by prior cannabis use (β=0.76, p=0.74) or the quantity of prior cannabis use (joints) (β=0.16, p=0.86) with an R^2=0.03. Moreover, the V_T of [^{18}F] FMPEP-d_2 in the striatum was not significantly predicted by prior cannabis use (β=2.90, p=0.23) or the quantity of prior cannabis use (joints) (β=-0.17, p=0.86) with an R^2=0.16.

Study 2

In a multiple linear regression, V_T of [^{11}C]MePPEP in the thalamus was not significantly predicted by prior cannabis use (β=-0.77, p=0.78) or the quantity of prior lifetime cannabis use (joints) (β=0.00, p=0.99) with an R^2=0.003. Similarly, the V_T of [^{11}C]MePPEP in the anterior cingulate was not significantly predicted by prior cannabis use (β=0.49, p=0.51) or the quantity of prior cannabis use (joints) (β=-0.56, p=0.05, with an R^2=0.14. In line with this, the V_T of [^{11}C]MePPEP in the hippocampus not significantly predicted by prior cannabis use (β=-0.33, p=0.67) or the quantity of prior cannabis use (joints) (β=0.14, p=0.28) with an R^2=0.01. Moreover, the V_T of [^{11}C]MePPEP in the striatum not significantly predicted by prior cannabis use (β=-0.22, p=0.36) or the quantity of prior cannabis use (joints) (β=-0.22, p=0.73) with an R^2=0.05.

eResults 8. Association Between CB1R Availability and Age

Study 1

In a linear regression, age did not significantly predict the V_T of [^{18}F] FMPEP-d_2 in the hippocampus (β=-0.07, p=0.64), thalamus (β=-0.06, p=0.38), anterior cingulate (β=-0.17, p=0.34) or the striatum (β=-0.17, p=0.34).

© 2019 Borgan F et al. *JAMA Psychiatry.*

Study 2

In a linear regression, age did not significantly predict the V_T of [^{11}C]MePPEP in the hippocampus (β=-0.27, p=0.42), striatum (β=-0.48, p=0.09), anterior cingulate (β=-0.26, p=0.40) or thalamus (β=-0.16, p=0.16).

eResults 9. Radiotracer Spatial Covariance

Study 1 & 2

The spatial covariance of the two radiotracers was compared using interregional correlation statistics as implemented by the NetPET package[12]. Both Krzanowski's tests on eigenvectors and eigenvalues did not show any statistical difference (peigenvector: 0.23, peigenvalue:0.19 lambda= 22.0, mu =28.9), supporting the fact that the distribution of the tracer uptake across brain tissue is similar for both patients and controls.

© 2019 Borgan F et al. *JAMA Psychiatry.*

eTable 2. Cannabinoid 1 Receptor Availability in Healthy Volunteers and First Episode Psychosis Patients Using a Whole Brain Voxelwise Analysis for Study 1 and 2

Study 1: $[^{18}F]$FMPEP-d_2 PET

Region	H	MNI	CS	T	Z	pFDR	pFWE	
Middle cingulate	L	-12 -12 42	55915	8.39	5.12	<0.001	<0.001	
Middle frontal gyrus	R	44 50 -2	1309	6.90	4.63	<0.001	<0.001	
Inferior orbital gyrus	R							
Superior frontal gyrus	R	16 52 9	1233	6.68	4.55	<0.001	<0.001	
Anterior cingulate	R							
Superior medial frontal gyrus								
Superior frontal gyrus	L	-10 42 46	614	6.25	4.38	<0.001	<0.001	
Middle temporal gyrus	R	45 -54 3	517	5.98	4.27	<0.001	<0.001	
Inferior temporal gyrus								
Anterior cingulate	L	-14 36 -2	461	5.74	4.17	<0.001	<0.001	
Inferior opercular gyrus	R	52 15 8	5.63	4.12	592	<0.001	<0.001	Inferior frontal pars
triangularis	R	39 22 22	100	5.48	4.05	0.003	0.027	
Middle temporal gyrus	L							
Inferior temporal gyus	L	-62 -27 -15	292	5.32	3.98	<0.001	0.001	

Study 2: $[^{11}C]$MePPEP PET

Region	H	MNI	CS	T	Z	pFDR	pFWE
Inferior temporal gyrus	R	40 8 -42	140	4.48	3.99	<0.001	<0.001

Voxel-wise analysis results from study 1 and 2 showing that cannabinoid 1 receptor availability was significantly lower in patients relative to controls.

H=hemisphere; MNI=Montreal Neurological Institute coordinates; R=right; L=left; CS=cluster extent (voxels); pFDR=p value following false discovery rate correction; pFWE= p value following family wise error correction.

© 2019 Borgan F et al. *JAMA Psychiatry.*

eFigure 1. CB1R Availability in the Hippocampus in Patients Relative to Healthy Volunteers in Study 1

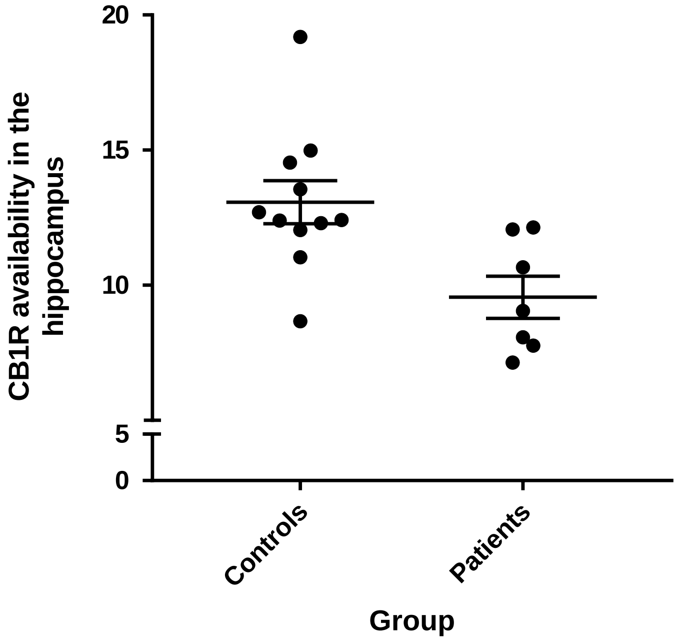

The distribution volume (V_T) of [18F] FMPEP-d2 was significantly lower in the hippocampus in patients with first episode psychosis relative to healthy volunteers in study 1. Mean and SEM values are shown.

© 2019 Borgan F et al. *JAMA Psychiatry.*

eFigure 2. CB1R Availability in the Striatum In Patients Relative to Healthy Volunteers in Study 1

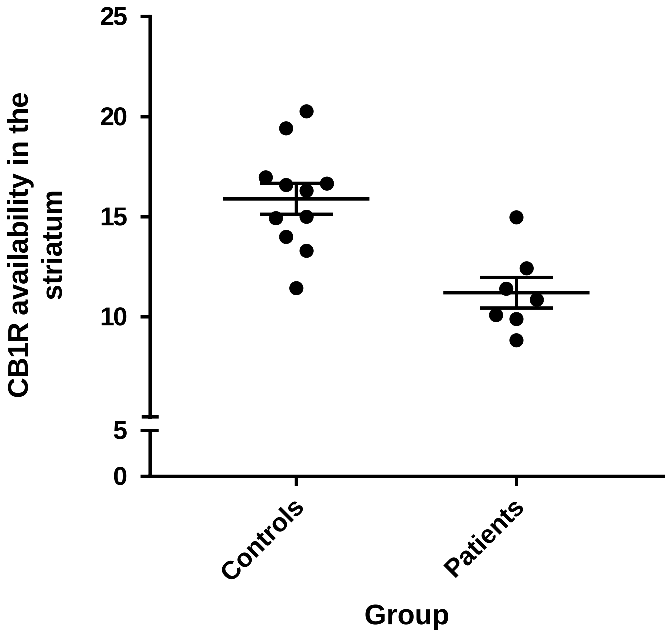

The distribution volume (V_T) of [18F] FMPEP-d2 was significantly lower in the striatum in patients with first episode psychosis relative to healthy volunteers in study 1. Mean and SEM values are shown.

© 2019 Borgan F et al. *JAMA Psychiatry.*

eFigure 3. CB1R Availability in the Anterior Cingulate Cortex in Patients Relative to Healthy Volunteers in Study 1

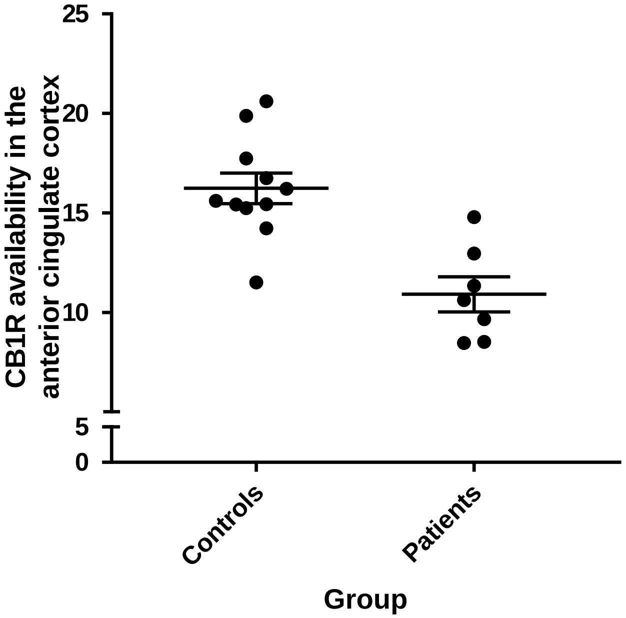

The distribution volume (V_T) of [18F] FMPEP-d2 was significantly lower in the anterior cingulate in patients with first episode psychosis relative to healthy volunteers in study 1. Mean and SEM values are shown.

© 2019 Borgan F et al. *JAMA Psychiatry.*

eFigure 4. CB1R Availability in the Thalamus in Patients Relative to Healthy Volunteers in Study 1

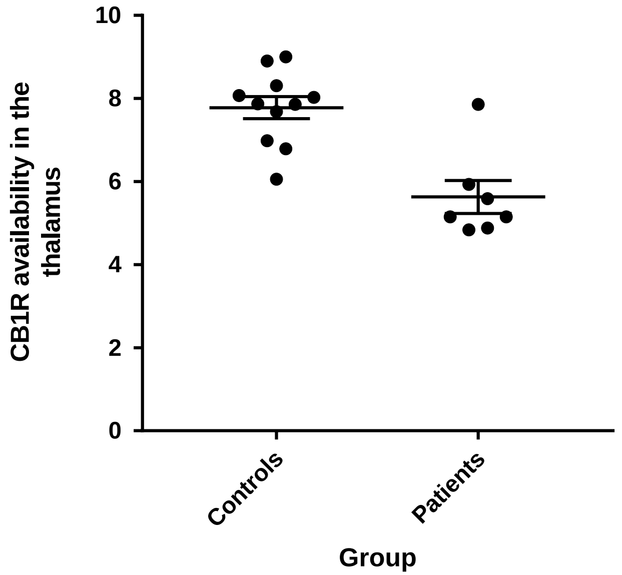

The distribution volume (V_T) of [18F] FMPEP-d2 was significantly lower in the thalamus in patients with first episode psychosis relative to healthy volunteers in study 1. Mean and SEM values are shown.

© 2019 Borgan F et al. *JAMA Psychiatry.*

eFigure 5. CB1R Availability in the Hippocampus in Patients Relative to Healthy Volunteers in Study 2

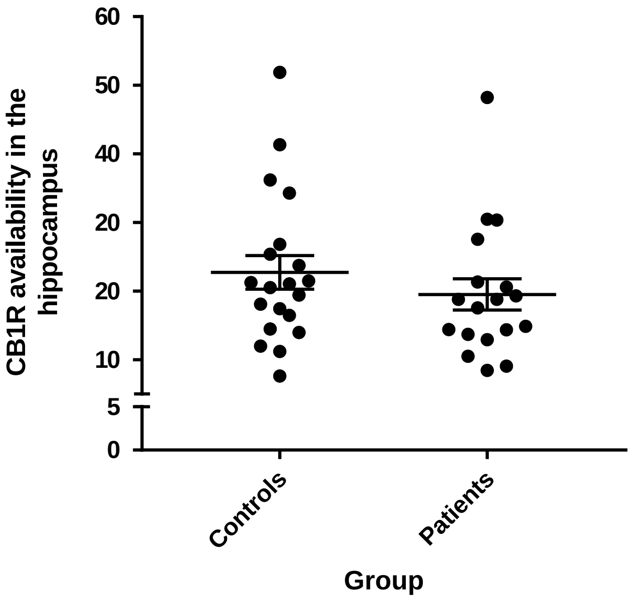

The distribution volume (V_T) of [11C]MePPEP was significantly lower in the hippocampus in patients with first episode psychosis relative to healthy volunteers in study 2. Mean and SEM values are shown.

© 2019 Borgan F et al. *JAMA Psychiatry.*

eFigure 6. CB1R Availability in the Striatum in Patients Relative to Healthy Volunteers in Study 2

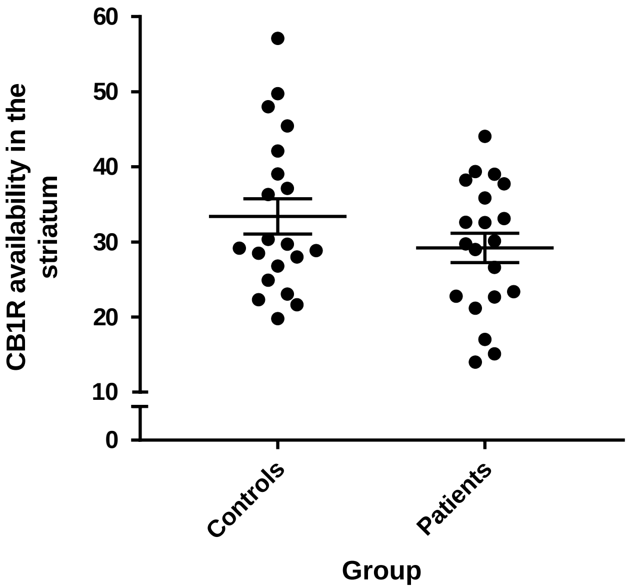

The distribution volume (V_T) of [11C]MePPEP was significantly lower in the striatum in patients with first episode psychosis relative to healthy volunteers in study 2. Mean and SEM values are shown.

© 2019 Borgan F et al. *JAMA Psychiatry.*

eFigure 7. CB1R Availability in the Anterior Cingulate Cortex in Patients Relative to Healthy Volunteers in Study 2

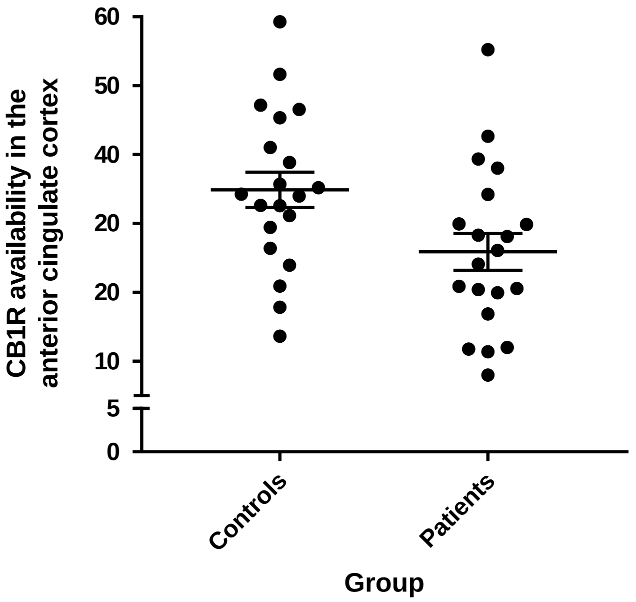

The distribution volume (V_T) of [11C]MePPEP was significantly lower in the anterior cingulate in patients with first episode psychosis relative to healthy volunteers in study 2. Mean and SEM values are shown.

© 2019 Borgan F et al. *JAMA Psychiatry.*

eFigure 8. CB1R Availability in the thalamus in Patients Relative to Healthy Volunteers in Study 2

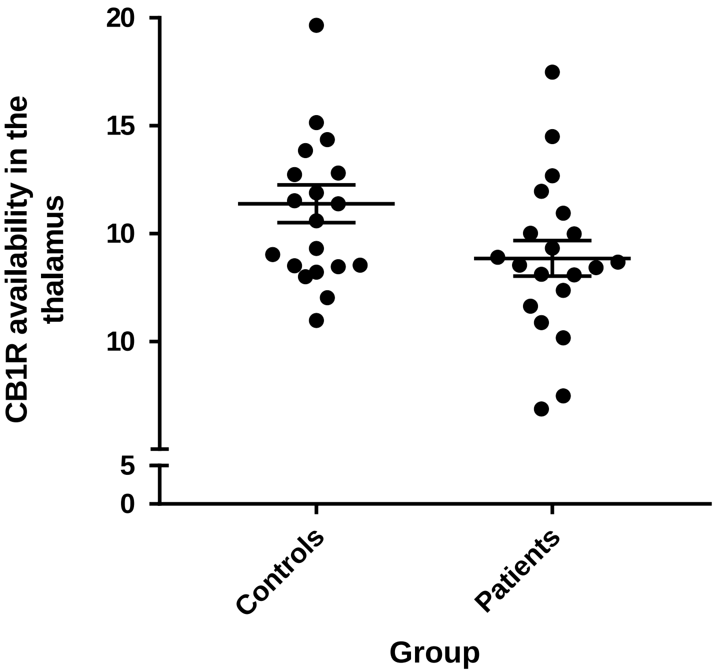

Legend: The distribution volume (V_T) of [11C]MePPEP was significantly lower in the thalamus in patients with first episode psychosis relative to healthy volunteers in study 2. Mean and SEM values are shown.

© 2019 Borgan F et al. *JAMA Psychiatry.*

eFigure 9. Whole-brain voxel-wise analysis showing CB1R availability in patients relative to controls from study 1

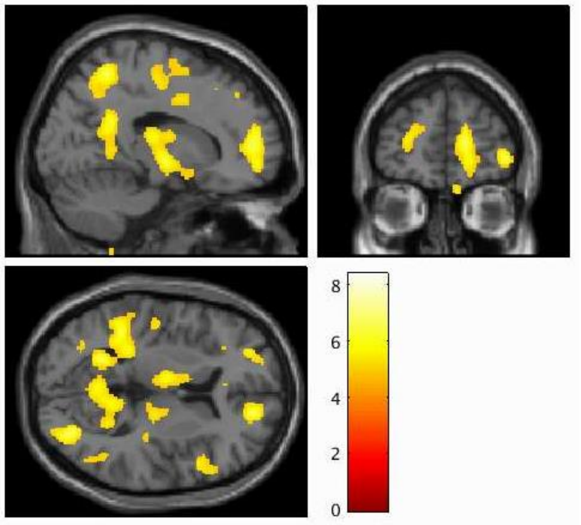

In a whole-brain voxel-wise analysis, patients relative to controls showed a decreased distribution volume of [^{18}F]FMPEP-d_2 PET in a cluster encompassing the right anterior cingulate cortex, right superior medial frontal gyrus (MNI coordinates: x=16, y=52, z=9), T=6.68, Z=4.55, pFWE<0.001.

© 2019 Borgan F et al. *JAMA Psychiatry.*

eFigure 10. Whole-brain voxel-wise analysis showing CB1R availability in patients relative to controls from study 2

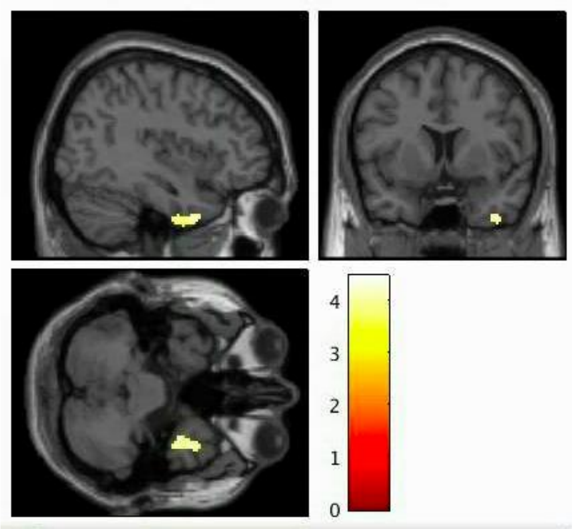

In a whole-brain voxel-wise analysis, patients relative to controls showed decreased distribution volume of [^{11}C]MePPEP PET in a cluster encompassing the inferior temporal gyrus and fusiform gyrus (MNI coordinates: x=40, y=8, z=-42), T=4.48, Z=3.99, pFWE<0.001

© 2019 Borgan F et al. *JAMA Psychiatry.*

References

1. Aalto, M., Alho, H., Halme, J. T. & Seppä, K. AUDIT and its abbreviated versions in detecting heavy and binge drinking in a general population survey. *Drug Alcohol Depend.* **103,** 25–29 (2009).
2. World Health Organization. Guidelines for Controlling and Monitoring the Tobacco Epidemic - WHO - OMS -. (1998). Available at: http://apps.who.int/bookorders/anglais/detart1.jsp?codlan=1&codcol=15&codcch=468. (Accessed: 11th March 2019)
3. Suvisaari, J. *et al.* Mental disorders in young adulthood. *Psychol. Med.* **39,** 287 (2009).
4. Quinn, C. A., Wilson, H., Cockshaw, W., Barkus, E. & Hides, L. Development and validation of the cannabis experiences questionnaire – Intoxication effects checklist (CEQ-I) short form. *Schizophr. Res.* **189,** 91–96 (2017).
5. Donohue, S. R. *et al.* Synthesis, Ex Vivo Evaluation, and Radiolabeling of Potent 1,5-Diphenylpyrrolidin-2-one Cannabinoid Subtype-1 Receptor Ligands as Candidates for In Vivo Imaging. *J. Med. Chem.* **51,** 5833–5842 (2008).
6. Yasuno, F. *et al.* The PET radioligand [11C]MePPEP binds reversibly and with high specific signal to cannabinoid CB1 receptors in nonhuman primate brain. *Neuropsychopharmacology* **33,** 259–69 (2008).
7. Riaño Barros, D. A. *et al.* Test-retest reproducibility of cannabinoid-receptor type 1 availability quantified with the PET ligand [^{11}C]MePPEP. *Neuroimage* **97,** 151–62 (2014).
8. Montgomery, A. J. *et al.* Correction of Head Movement on PET Studies: Comparison of Methods. *J Nucl Med* **47,** 1936–1944 (2006).
9. Terry, G. E. *et al.* Imaging and quantitation of cannabinoid CB1 receptors in human and monkey brains using (18)F-labeled inverse agonist radioligands. *J. Nucl. Med.* **51,** 112–20 (2010).
10. Hammers, A. *et al.* Three-dimensional maximum probability atlas of the human brain, with particular reference to the temporal lobe. *Hum. Brain Mapp.* **19,** 224–247 (2003).
11. Coodin, S. Body Mass Index in Persons with Schizophrenia. *Can. J. Psychiatry* **46,** 549–555 (2001).
12. Veronese, M. *et al.* Covariance statistics and network analysis of brain PET imaging studies. *Sci. Rep.* **9,** 2496 (2019).

© 2019 Borgan F et al. *JAMA Psychiatry.*
